# Compartmental and Temporal Dynamics of Chronic Inflammation and Airway Remodelling in a Chronic Asthma Mouse Model

**DOI:** 10.1371/journal.pone.0085839

**Published:** 2014-01-21

**Authors:** Mohammed Alrifai, Leigh M. Marsh, Tanja Dicke, Ayse Kılıç, Melanie L. Conrad, Harald Renz, Holger Garn

**Affiliations:** 1 Institute of Laboratory Medicine and Pathobiochemistry - Molecular Diagnostics, Medical Faculty, Philipps University Marburg, Universities of Giessen and Marburg Lung Center (UGMLC), Member of the German Center for Lung Research, Marburg, Germany; 2 Ludwig Boltzmann Institute for Lung Vascular Research, Graz, Austria; Research Center Borstel, Germany

## Abstract

**Background:**

Allergic asthma is associated with chronic airway inflammation and progressive airway remodelling. However, the dynamics of the development of these features and their spontaneous and pharmacological reversibility are still poorly understood. We have therefore investigated the dynamics of airway remodelling and repair in an experimental asthma model and studied how pharmacological intervention affects these processes.

**Methods:**

Using BALB/c mice, the kinetics of chronic asthma progression and resolution were characterised in absence and presence of inhaled corticosteroid (ICS) treatment. Airway inflammation and remodelling was assessed by the analysis of bronchoalveolar and peribronichal inflammatory cell infiltrate, goblet cell hyperplasia, collagen deposition and smooth muscle thickening.

**Results:**

Chronic allergen exposure resulted in early (goblet cell hyperplasia) and late remodelling (collagen deposition and smooth muscle thickening). After four weeks of allergen cessation eosinophilic inflammation, goblet cell hyperplasia and collagen deposition were resolved, full resolution of lymphocyte inflammation and smooth muscle thickening was only observed after eight weeks. ICS therapy when started before the full establishment of chronic asthma reduced the development of lung inflammation, decreased goblet cell hyperplasia and collagen deposition, but did not affect smooth muscle thickening. These effects of ICS on airway remodelling were maintained for a further four weeks even when therapy was discontinued.

**Conclusions:**

Utilising a chronic model of experimental asthma we have shown that repeated allergen exposure induces reversible airway remodelling and inflammation in mice. Therapeutic intervention with ICS was partially effective in inhibiting the transition from acute to chronic asthma by reducing airway inflammation and remodelling but was ineffective in preventing smooth muscle hypertrophy.

## Introduction

Human bronchial asthma is a chronic inflammatory disease of the airways that is characterized by chronic airway inflammation, airway hyperresponsiveness (AHR), and airway remodelling [1]–[3]. Hallmarks of the structural changes in the airways include goblet cell hyperplasia, collagen deposition and increased smooth muscle mass [4], [5]. Remodelling is thought to arise either from excessive repair processes or the failure to resolve allergen driven inflammation [6]. Current anti-inflammatory treatment of asthma is predominately based on the use of inhaled corticosteroids (ICS). Although these drugs are highly effective in preventing life threatening consequences of asthma [7], their effect is limited in modulating airway remodelling [8]. The synthetic glucocorticoid, budesonide is a well-established compound used locally to treat allergic diseases and asthma [9]. The therapeutic potential of budesonide has extensively been studied in models of acute allergic inflammation but only a few studies have investigated efficacy on established airway remodelling and chronic asthma [10], [11].

The majority of animal studies are based on models of acute allergic airway inflammation [12]. Although these models induce a strong acute allergic inflammation, they do not develop further major characteristics of the human disease such as chronic airway remodelling. exemplary collagen deposition or smooth muscle thickening [13]. Alternative models have since been developed that more closely reflect the pathological changes observed in patients [3], [14], [15]. Such models are required to study novel intervention methods in a therapeutic rather than a prophylactic setting as investigated in acute asthma models [16]. Although the development of allergen-induced airway inflammation and remodelling has been extensively examined, few studies have addressed the resolution of allergic inflammation [6].

We here have characterised the inflammatory and remodelling events that contribute to the transition from acute to chronic experimental asthma. Furthermore, we have studied the impact of ICS treatment during this transition phase, to specifically identify steroid-sensitive and -resistant pathways. The reversibility of remodelling has been examined following a period of ICS therapy and in the optimal situation of allergen avoidance.

## Materials and Methods

### Animals

Female BALB/c mice aged 6–8 weeks were purchased from Harlan Winkelmann (Hannover, Germany) and were maintained under pathogen-free conditions in isolated ventilated cages with 12 hour light/dark cycles. Water and ovalbumin (OVA)-free diet were supplied ad libitum. All mouse experiments met German and international guidelines and were approved by the Regierungspraesidium Giessen, and all measures were taken to keep animal suffering to a minimum.

### Experimental animal models

**Figure 1:**
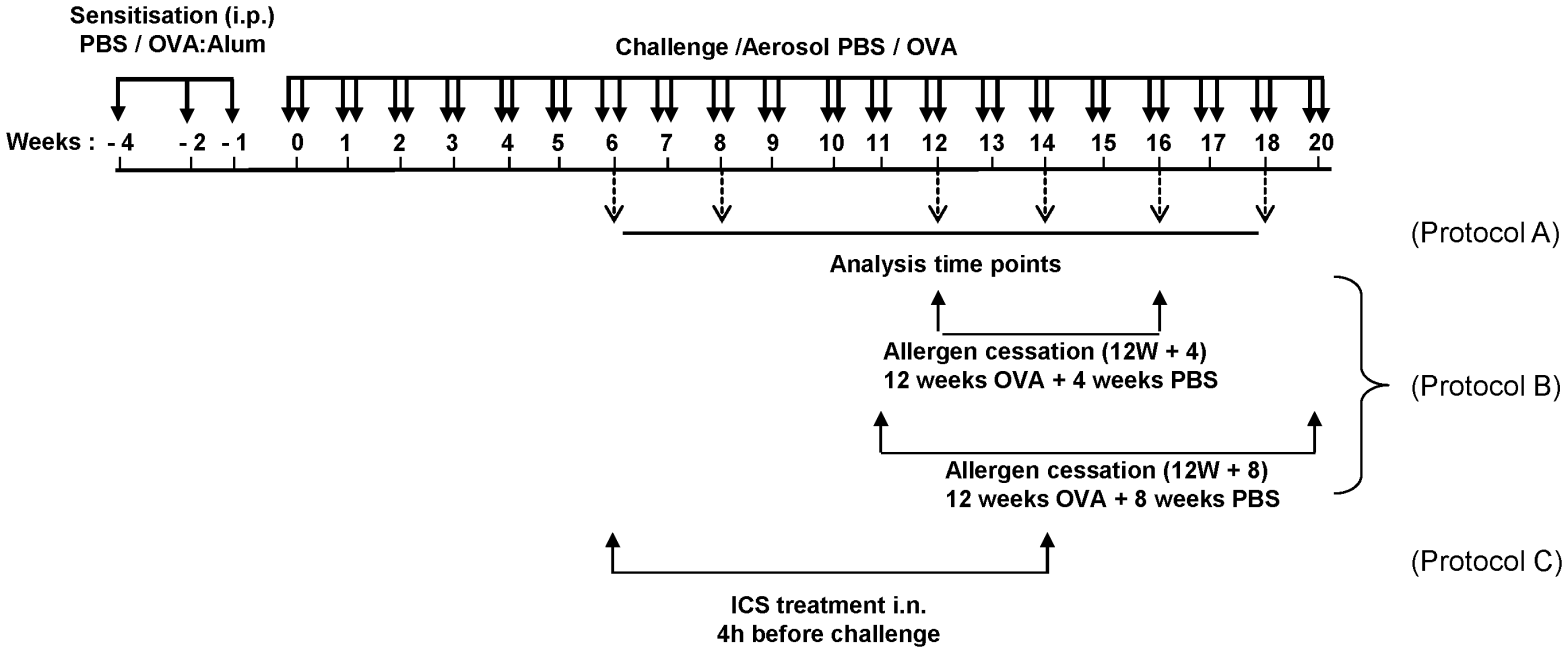
Schematic representation of treatment protocol. Mice were sensitised to OVA by three intraperitoneal (i.p.) injections on days 0, 14 and 21 with OVA absorbed to alum. Mice were sensitised and challenged with aerosolised OVA twice weekly for up to 18 weeks as indicated. Control mice were sensitised and challenged with PBS. Analysis was performed after 6, 8, 12, 14, 16 and 18 weeks of challenge (Protocol A). To investigate the resolution of airway inflammation and remodelling, OVA was replaced with PBS after 12 weeks for either 4 or 8 weeks (Protocol B). In a third study, inhaled corticosteroids (ICS) were given intranasally (i.n.) starting after 6 weeks of OVA challenge for 4 weeks (Protocol C).

Mice were sensitised to OVA by three intraperitoneal (i.p.) injections of 10 µg OVA grade VI (Sigma, Hamburg, Germany) adsorbed to 1.5 mg Al(OH)_3_ (Pierce, Rockford, IL, USA) diluted in 200 µl phosphate-buffered saline (PBS). Mice were challenged with OVA (grade V) aerosol (1% wt/vol in PBS) twice a week on 2 consecutive days over a period of up to 18 weeks, control groups received PBS. One group of mice was additionally treated with 50 µl of 200 µg/ml budesonide (Astra Zeneca, Lund, Sweden) intranasally (i.n.) 4 hours before each OVA challenge. Group composition is illustrated in Figure 1. As no differences were detected between control mice in different time points, data is shown only for one PBS group. In an acute model of experimental asthma, OVA sensitised mice were challenged 3 times by OVA aerosol (Figure S2). All experiments were performed once with a group size of 6–8 mice treated in parallel in accordance to German animal ethic regulations. Each group was coded and analysed by an investigator blinded to the experimental conditions.

### Bronchoalveolar lavage fluid (BALF) and differential cell counts

Mice were sacrificed and BALF was obtained using 1 ml PBS containing protease inhibitor cocktail (Roche, Mannheim, Germany) as described previously [17]. Cytospin preparations were prepared and stained with Diff-Quick (Merz & Dade AG, Dudingen, Switzerland). Differential counts were performed based on morphologic and histologic criteria (300 cells counted).

### Lung histology and immunohistochemistry

Lungs were fixed ex situ with 6% (wt/vol) paraformaldehyde via the trachea, removed and stored in 6% paraformaldehyde. Independent uniform random (IUR) sections were then prepared in order to obtain a representative collection of lung tissue samples [18]. In brief, the lungs were first embedded in 2% agar, 2 mm thick sections were cut in a random plane generating isotropic sections; these were then randomly transferred to embedding cassettes for paraffin embedding. Three µm lung tissue sections were then cut and stained by immunohistochemistry or with haematoxylin and eosin (H&E), sirius red staining or periodic acid-Schiff reagent (PAS). For immunohistochemistry, sections were stained with rabbit anti-SMA antibody (RB-9010–P, Lab vision, USA) or goat anti-CD3 antibody (sc-1127, Santa Cruz Biotechnology, UK) in PBS with 3% milk powder overnight. Primary antibodies were detected by first incubation with biotinylated anti-rabbit or anti-goat antibodies (Vector, Peterborough, UK) for 1 hour followed by 30 min incubation with Horseradish peroxidise-strepavidin and finally DAB substrate (Vector) for 10 min in the dark. The sections were then rinsed briefly with distilled water and then counterstained for 1 min in haematoxylin.

### Measurement of cytokines in bronchoalveolar lavage fluid (BALF)

IL-4, IL-5, IL-13, IFN-γ (BD Biosciences, Heidelberg, Germany) and TGF-β (R&D Systems, Minneapolis, MN, USA) were measured by ELISA in cell-free lavage fluids according to the manufacturers' protocol. The detection limit for each cytokine was 10 pg/ml and 20 pg/ml for TGF-β.

### Quantitative morphology

H&E-stained tissue sections were viewed and random images collected under 20× objective. Degree of inflammation was expressed as a peribronchial airway inflammation score (0, normal; 1, few inflammatory cells; 2, one to two cell layer ring of inflammatory cells; 3, three to four cell layer ring of inflammatory cells and 4, more than four layer ring of inflammatory cells) [17]. H&E and CD3 immunohistochemical stained lung sections were selected by random sampling (40–50 images) using the 40× objective. The number of eosinophils and CD3-positive cells were quantified and expressed as cell number per field.

PAS-stained sections were viewed and random images collected under 20× objective. The fraction of the analysed basal membrane covered by goblet cells was then evaluated. Inflammation and goblet cells were quantified using a PC-based Olympus light microscope BX51 equipped with a Cell-F System (Olympus, Hamburg, Germany). Paraffin sections stained with sirius red, anti-SMA, were used to quantify changes in airway collagen deposition, smooth muscle cell layer thickening, respectively, using the BX51 microscope equipped with a CAST-Grid System (Visiopharm, Hoersholm, Denmark) [3]. All sections were delineated and the fields of view analysed (at 400x) were automatically defined according to systematic uniform random sampling, 150 random samples (∼30% of total lung tissue area and thus representing all parts of the airway tree) were taken of each section. The arithmetic mean thickness (*T_comp_*) was determined as the volume of the respective component, determined by counting all points intercepting the airway epithelium and Sirius Red- and α-SMA-positive components, respectively [18], [19]. Results were referred to the reference surface determined by counting all intersections with the airway epithelial basal membrane. The arithmetic mean thickness was calculated according to the formula: *T_comp_  =  L(P) ×* Σ *P_comp_/*(2× Σ *I_bi_*) [19]. *L(P)* is the line length per test point, *P_comp_*, the number of points hitting the respective component and *I_bl_* the number of intersections between the test line and the epithelial basal membrane.

### Statistical analysis

Graphing and statistical analysis of normally distributed data was performed using Graph Pad Prism 5 (La Jolla, CA, USA). Data are expressed as mean ± SEM or as mean and percentiles. Statistical significance was determined using one-way ANOVA with Tukey's Multiple Comparison Test (for multiple group comparison) or the Student's unpaired t-test for two group comparison. Statistical significance was referred to as follows *p≤0.05, **p≤0.01, ***p≤0.001.

## Results

### Chronic allergen challenge results in prolonged airway inflammation and remodelling

**Figure 2:**
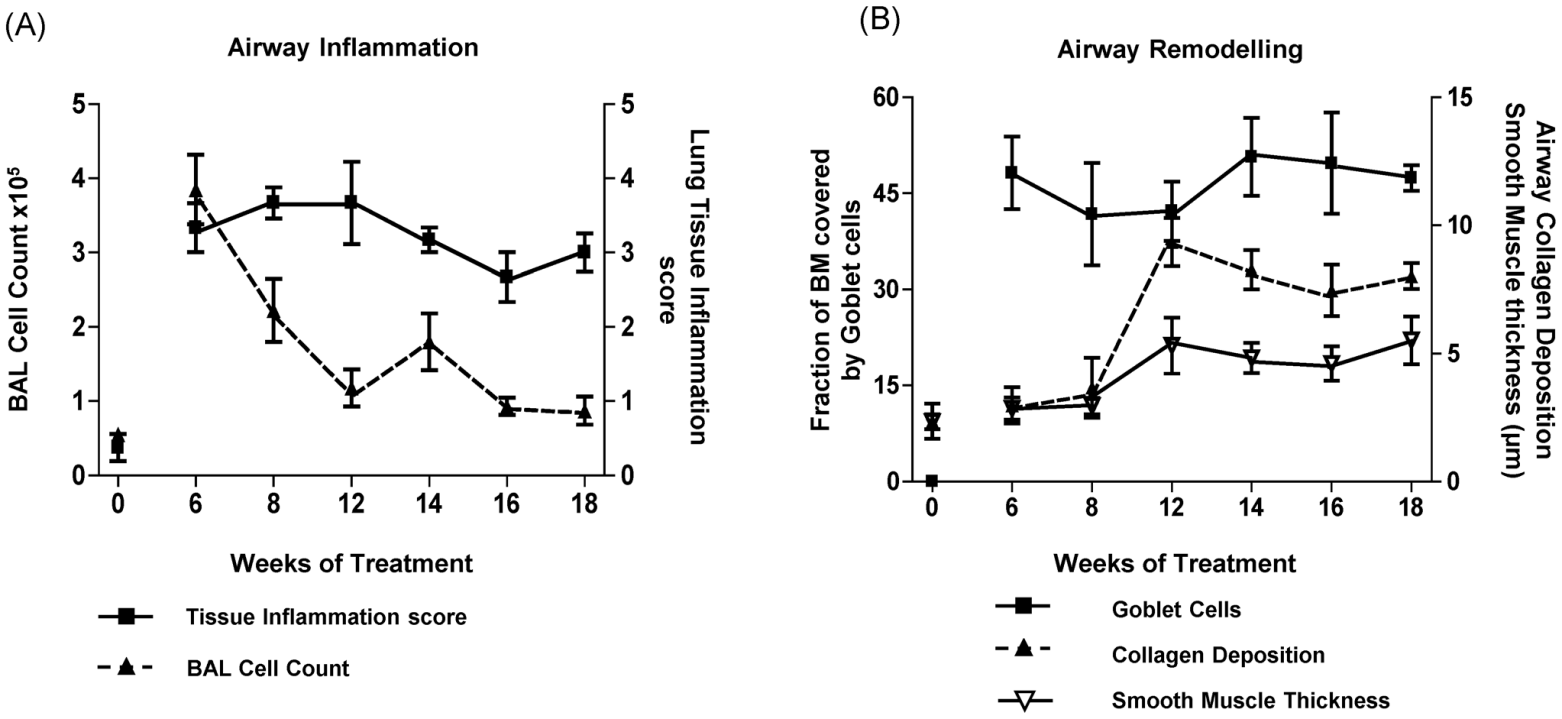
Chronic allergen exposure induces airway inflammation and remodelling. Mice were challenged with OVA for up to 18 weeks (Figure 1, Protocol A) and analysed for airway inflammation, as determined by (A) BALF cell counts and morphometric quantification of peribronchial inflammation. (B) Airway remodelling was determined by quantification of goblet cell hyperplasia, subepithelial collagen deposition and smooth muscle thickening. Data points represent means ± SEM of n = 6–8 animals per group.

The kinetics of airway inflammation was investigated using a chronic mouse model of experimental asthma by challenging mice with aerosolised OVA over an eighteen week period (Figure 1, protocol A). In the bronchoalveolar lavage fluid (BALF), peak cell infiltration was observed at 6 weeks of OVA challenge, which steadily decreased until 12 weeks and then remained at almost baseline levels (Figure 2A and Figure S1). In contrast, the high level of peribronchial tissue inflammation observed at six weeks, persisted throughout all analysed time points (Figure 2A).

Hallmarks of airway remodelling are goblet cell hyperplasia, thickening of the smooth muscle cell layer and extracellular matrix deposition. To quantify changes in airway remodelling immunohistochemical analysis in combination with stereological quantification was performed. Comparison of PAS-stained lung sections from chronically OVA treated mice revealed that ∼45% of all cells lining the bronchial airways throughout the whole observation period were goblet cells. Advanced structural changes, such as collagen deposition and smooth muscle thickening, in the airways were not detected until eight weeks of OVA exposure as determined by collagen and α-SMA staining, respectively. Between 8 and 12 weeks the amount of airway collagen rapidly increased by approximately 3 fold and then remained constant. Thickening of the smooth muscle layer was less pronounced than collagen deposition, but increased approximately two fold between 6 and 12 weeks (Figure 2B). Together this data shows that in this mouse model of chronic experimental asthma, continued allergen exposure is associated with prominent and persistent airway inflammation and structural alterations.

### Cessation of allergen exposure reverses airway inflammation and remodelling

**Figure 3:**
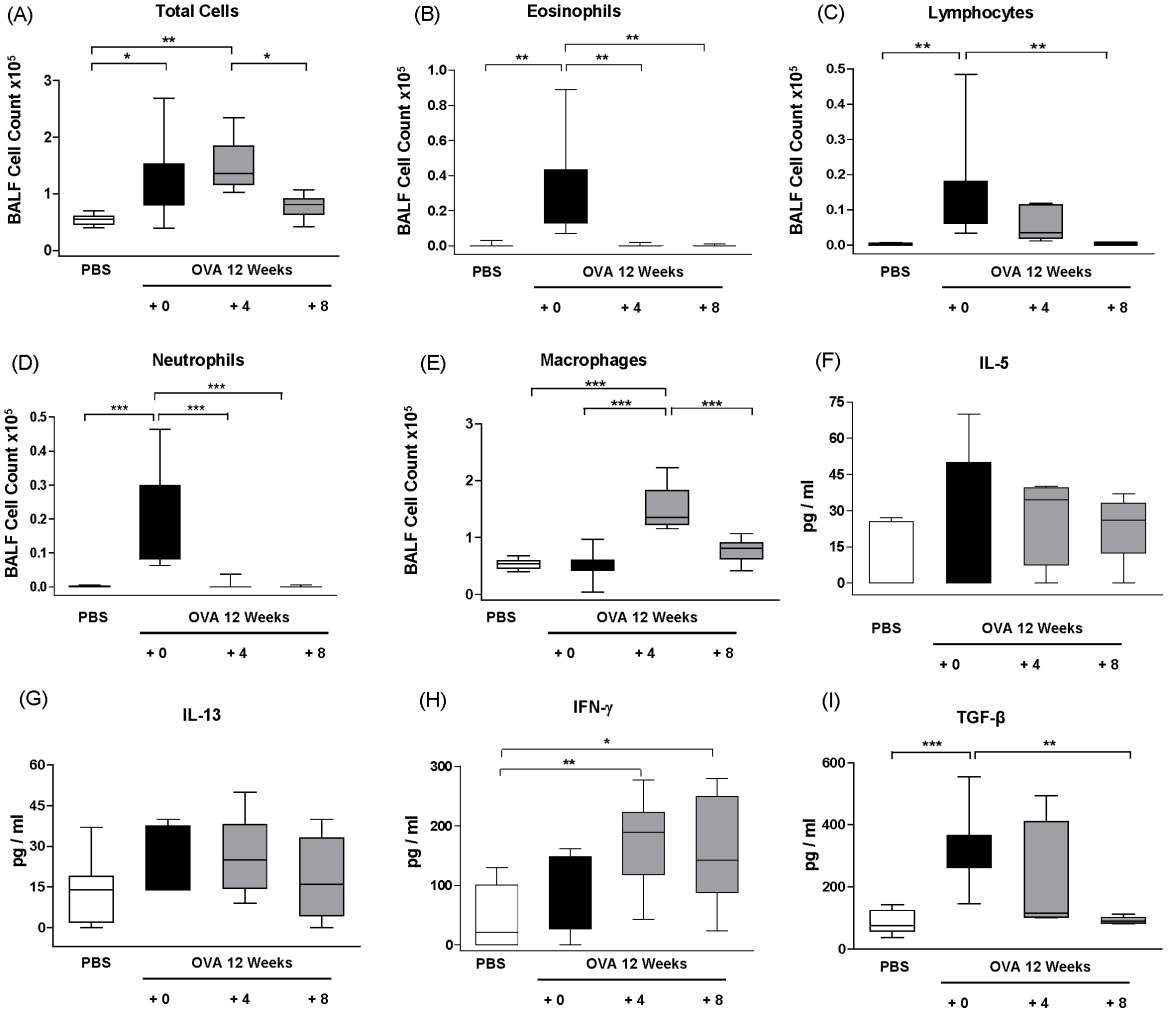
Resolution of BALF inflammation and cytokine profile following allergen cessation. Mice were challenged with OVA twice weekly for 12 weeks to establish features of chronic asthma; OVA challenge was then discontinued and replaced with PBS for 4 or 8 weeks (Protocol B). BALF inflammation was analysed after 12 (+ 0), 16 (+ 4) or 20 (+ 8) weeks of challenge. The results are presented as box and whisker plots showing mean and percentiles of n = 6–8 animals per group, *p≤0.05, **p≤0.01, ***p≤0.001.

To mimic the situation of effective allergen avoidance, it was investigated how allergen cessation would affect established airway remodelling and inflammation. Following 12 weeks of OVA challenge mice exhibited robust airway inflammation and fully established remodelling, including thickening of the smooth muscle layers and increased deposition of collagen. Therefore, this time point was chosen as the reference point for the chronic situation and used to investigate the effects of allergen cessation over the following eight week period. After four weeks of resolution, total BALF cell numbers remained constant but the numbers of eosinophils and neutrophils decreased to baseline levels (Figure 3A,B,D). The number of lymphocytes in the BALF resolved much slower, returning to PBS levels after eight weeks together with total BALF cell counts (Figure 3A,C). Interestingly, the number of alveolar macrophages first significantly increased then decreased to PBS levels after eight weeks of resolution (Figure 3E). Before allergen cessation and during the eight week resolution phase, the levels of IL-5 in the BALF were indistinguishable from the PBS group (Figure 3F). The levels of IL-13 were increased at the chronic reference point (12 weeks of OVA challenge) then decreased during resolution period, however, these changes did not reach statistical significance (Figure 3G). In contrast, the levels of IFN-γ were increased during the resolution phase (Figure 3H). High levels of the pro-remodelling cytokine TGF-β were detected in the BALF at 12 weeks of OVA challenge, which then returned to baseline levels after 8 weeks of allergen cessation (Figure 3I).

**Figure 4:**
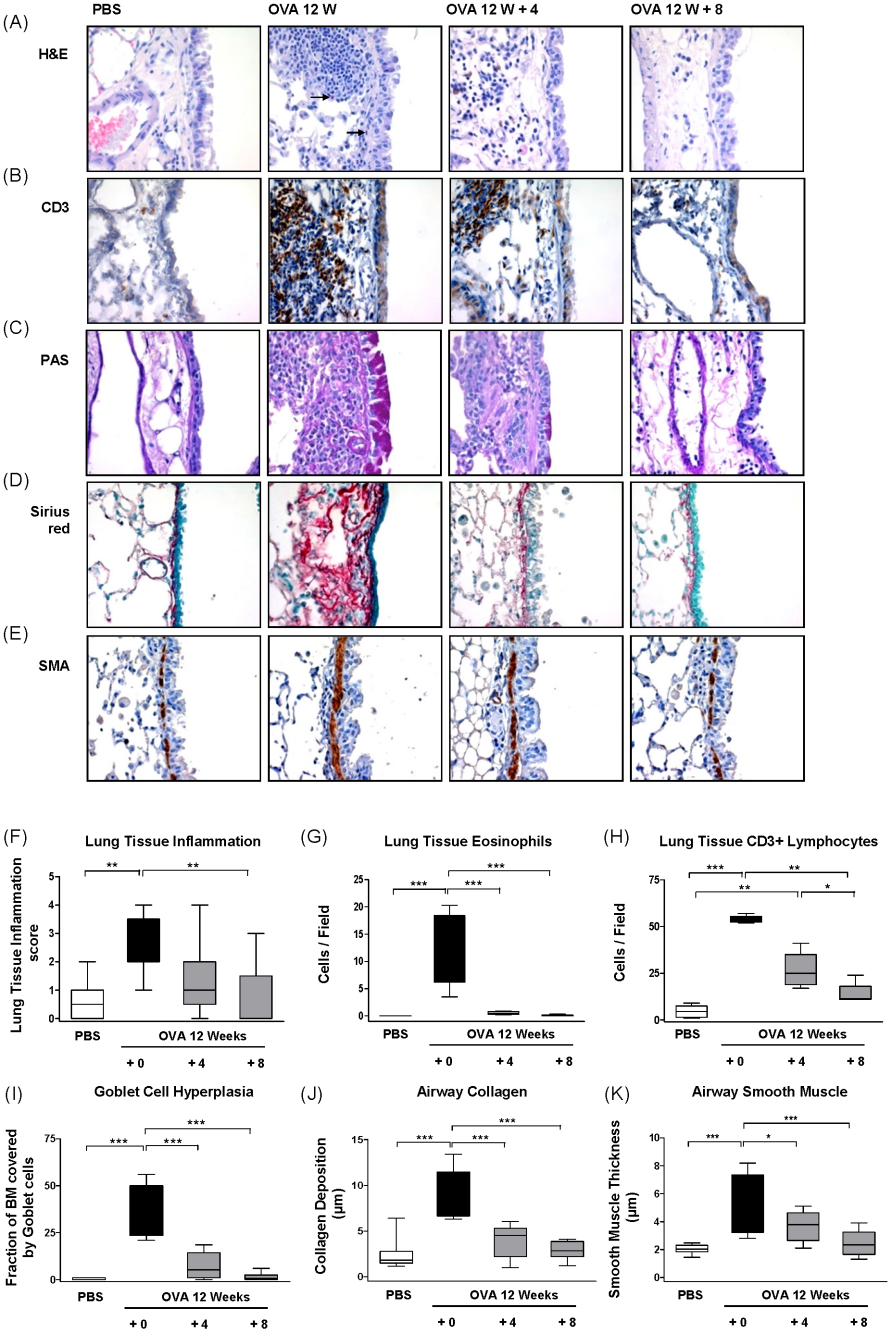
Resolution of airway tissue inflammation and remodelling following chronic allergen exposure. Chronic asthma was established by challenging OVA sensitized mice for 12 weeks twice weekly. OVA challenge was then discontinued and replaced with PBS for four or eight weeks. Outcome measurements were made at either 12 (+ 0), 16 (+ 4) or 20 (+ 8) weeks of challenge (Figure 1, Protocol B). (A) Representative photomicrographs of haematoxylin & eosin (H&E) stained sections (arrows indicate eosinophils, (B) immunohistochemical staining of CD3, (C) periodic acid-Schiff (PAS), (D) Sirius Red staining and (E) immunohistochemical staining of smooth muscle actin (SMA). (F) Histological quantification of lung inflammation, (G) eosinophil numbers as determined by morphological criteria in H&E stained lung sections, (H) CD3^+^ lymphocytes, (I) fraction of basal membrane (BM) covered by goblet cells, (J) collagen deposition and (K) SMA thickness. Box and whisker plots show mean and percentiles of n = 6–8 animals per group, *p≤0.05, **p≤0.01, ***p≤0.001.

Tissue inflammation and airway remodelling were assessed by immunohistochemical staining in combination with stereological quantification (Figure 4). The high level of lung tissue inflammation observed before allergen cessation slowly decreased and returned to baseline levels after the 8 week resolution period (Figure 4F). While lung tissue eosinophils rapidly disappeared (Figure 4G), many CD3^+^ T lymphocytes were still present after 4 weeks and only reduced after 8 weeks of allergen cessation (Figure 4H). Before resolution ∼40% of the airway epithelium consisted of goblet cells, which decreased to ∼8% after 4 weeks of allergen cessation then further decreased to baseline levels after 8 weeks of resolution. Airway collagen deposition exhibited a similar trend, rapidly decreasing in thickness during the initial four weeks of allergen avoidance and finally resolving at eight weeks. On the other hand, smooth muscle thickening was much slower to resolve, requiring the full eight weeks to return to untreated levels (Figure 4I-K). Together this data shows that the resolution of inflammation and remodelling are highly dynamic processes that occur with different kinetics for individual parameters.

### Inhaled corticosteroids protect against the full establishment of airway remodelling during development of chronic asthma

It was next investigated whether therapeutic intervention could interfere with the development of advanced airway remodelling. The efficacy of ICS treatment at the given dose was first determined using an acute model of experimental asthma (Figure S2). As expected, acute OVA challenge induced a strong recruitment of inflammatory cells into the BALF, predominately consisting of eosinophils as well as high levels of goblet cell hyperplasia. ICS administration significantly attenuated the OVA-induced asthma phenotype. However, treatment did not completely attenuate experimental asthma manifestation, as the numbers of eosinophils and goblet cells remained significantly higher than in control mice (Figure S2). The effect of ICS on the development and progression of airway remodelling was then examined in the chronic model; treatment started at a time when initial remodelling processes are observed (Figure 2; week 6) and then continued during the period of reinforcement and full establishment of remodelling (Figure 2; week 14).

**Figure 5:**
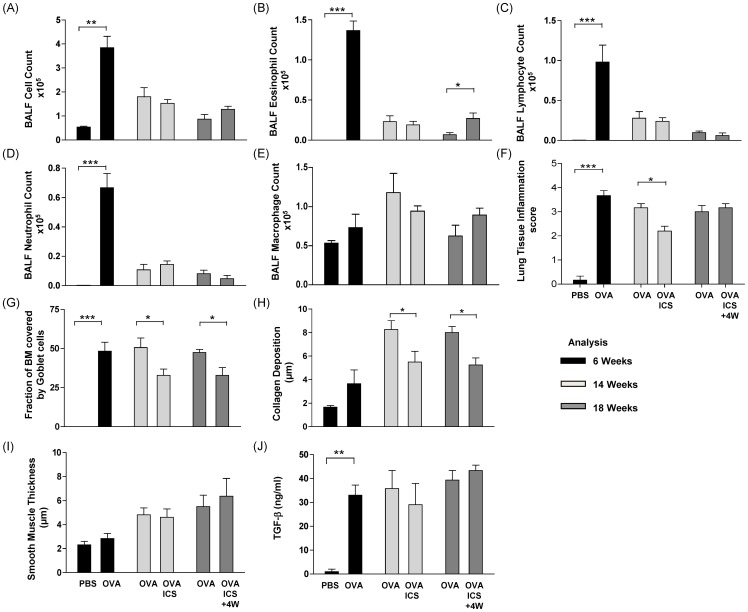
Inhaled corticosteroids attenuate some but not all characteristics of chronic asthma. Mice were sensitised and challenged for six weeks with PBS or OVA (black bars). OVA treatment was continued for another eight weeks (light grey bars), with or without parallel treatment with inhaled corticosteroids (ICS). OVA treatment was then continued for another 4 weeks without ICS application (dark grey bars) (Protocol C). Data are presented as mean ± SEM of n = 6–8 animals per group, *p≤0.05, **p≤0.01, ***p≤0.001.

Prior to ICS therapy (week 6), OVA challenge induced a strong bronchoalveolar and tissue inflammation, recruiting eosinophils, neutrophils and lymphocytes to the BALF (Figure 5, black bars). Treatment of mice with ICS for 8 weeks did not alter the level of bronchoalveolar inflammation (Figure 5A–E, light grey bars), however, significantly reduced tissue inflammation (Figure 5F, light grey bars) compared to mice challenged with OVA alone. ICS therapy also diminished goblet cell numbers and collagen deposition, but had no effect on smooth muscle thickening (Figure 5G–I, light grey bars).

To investigate whether this protection persisted following discontinuation of ICS, mice were further exposed to OVA for another four weeks in the absence of corticosteroids. Mice previously treated with ICS exhibited increased eosinophil numbers in the BALF as compared to mice that never received ICS, (Figure 5B, dark grey bars). However, mice that were treated with ICS maintained lower goblet cell numbers and reduced collagen deposition compared to mice that did not receive ICS (Figure 5G, H, dark grey bars). Analysis of total TGF-β levels in the BALF revealed a slight but non-significant decrease after treatment with ICS (light grey bars), Four weeks post ICS (dark grey bars) similar TGF-β levels were observed in steroid naïve and ICS treated mice (Figure 5J). Together, this data shows that ICS confers some protection from advanced remodelling during the transition from the acute to the chronic phase. However, some beneficial effects of ICS are lost in the case of subsequent allergen exposure.

## Discussion

Pronounced airway remodelling is a hallmark of chronic asthma and is characterised by goblet cell hyperplasia, deposition of extracellular matrix components and thickening of the smooth muscle layer. The presence of advanced airway remodelling is associated with a poorer clinical prognosis and is therefore, considered an important therapeutic target [20], [21]. Unfortunately, current anti-inflammatory therapeutic strategies including corticosteroids, while effective for reducing inflammation are less successful in treating structural alterations in airway remodelling [11]. Furthermore, the reversibility of airway remodelling is still unclear; it is not fully understood whether cessation of allergen exposure can lead to the full resolution of established remodelling [14]. To address these open questions, we have utilised a mouse model of chronic asthma which exhibits pronounced airway remodelling at 12 weeks of aerosol allergen exposure and is maintained throughout the entire challenge period of 18 weeks.

The maintenance of chronic asthma and tissue inflammation was accompanied by decreased bronchoalveolar inflammation but persistence of tissue inflammation. The low levels of eosinophils and lymphocytes present within the BALF at the later time points during allergen challenge is consistent with previous studies [3], [22]. This data also supports clinical observations by Persson *et al*. who described that a decrease in inflammatory BALF cells but the persistence of lung tissue inflammation is an index of worse outcome in asthma [23]. These data demonstrate that decreased inflammatory cell numbers in the BALF but maintenance of tissue inflammation correlates with the progression of chronic allergic asthma and is independent from resolution. Although compartmentalisation of airway-inflammation seems to be a critical step during the transition from an acute to a chronic phenotype, the underlying molecular mechanisms which regulate compartmentalisation of inflammatory cells are still not known. It is likely that selective and spatial recruitment processes direct this phenotype, which includes the expression of adhesion molecules, chemokines and/or chemokine receptors. It has been reported that prolonged allergen challenge can lead to immune tolerance and loss of inflammation [24]. However, in this and other studies it has been demonstrated that prolonged allergen exposure results in persistent goblet cell hyperplasia and chronic tissue inflammation [22]. The discrepancy between these reports is most likely due to the use of different mice strains; C57BL/6 versus BALB/c as used in our study [25]–[27], which indicates an underlying genetic component in asthma susceptibility and recovery.

In mice with fully established airway inflammation and remodelling, allergen cessation (four weeks) resulted in a rapid decrease in inflammatory cells such as eosinophils and neutrophils from the BALF. In contrast, macrophage numbers revealed a different kinetics initially increasing in numbers before returning to baseline levels. This temporary increase in macrophage numbers adds further support to the important role of this cell type in the resolution of inflammation [5], [28]. Allergen cessation resulted in a rapid decrease in goblet cell numbers, which is in line with observations made by other investigators [29]–[31]. Our study expands on these investigations by performing comprehensive analysis of both inflammatory and remodelling parameters. It has been proposed that the cessation of allergen exposure does not completely attenuate airway remodelling [1], [14], [31], [32]. In the studies by McMillan *et al.* and by Kumar *et al*. four weeks of allergen cessation was not sufficient to fully resolve airway remodelling [14], [31]. This observation was confirmed by the results of our study, however, prolongation of the resolution period to eight weeks completely attenuated lung tissue inflammation and fully reversed airway remodelling. Together this supports the notion that continued allergen exposure is required for the persistence of allergic airway inflammation and remodelling, and that avoidance of allergen exposure could ameliorate airway inflammation and remodelling.

The extensive airway remodelling at twelve weeks of OVA-challenge correlated with high levels of TGF-β in the BALF. TGF-β has important roles in mediating remodelling by inducing the production of extracellular matrix proteins and cell proliferation. It has been shown that TGF-β has a significant role in pulmonary fibrosis [33]. Additionally increased TGF-β expression has been observed in asthmatic patients, which correlated with sub-epithelial fibrosis [34]–[36]. Furthermore, in our study the decreasing level of TGF-β in BALF following allergen cessation also correlated with the resolution of airway remodelling, which further indicates the important role of this cytokine in remodelling and resolution. The increased IFN-γ levels observed during resolution phases may also serve to further antagonise the pro-fibrotic effects of TGF-β [37].

ICS are the mainstay of asthma therapy in humans [38]. Studies in mice have predominately focused on the effects of ICS in acute asthma models [9], [39], [40]. We have here investigated the effects of ICS during the establishment of airway remodelling. The experimental protocol closely mimics the clinical situation, in which patients suffer from acute allergic asthma symptoms at the starting point of treatment. The data from the acute model confirmed the efficacy of the ICS treatment and is consistent with other studies [9], [39], [40]. Applying ICS during the transition from acute to chronic asthma, resulted in lower lung tissue inflammation, goblet cell hyperplasia and collagen deposition. ICS however did not alter allergen induced smooth muscle thickening. Together these results indicate that despite ICS OVA sensitisation is retained and that ICS delay some but not all characteristics of chronic remodelling.

Similar observations were reported in a chronic OVA-induced asthma model when budesonide was given for four weeks following allergen cessation, however in this case no differences in collagen deposition and smooth muscle mass were observed [2]. Similar results have been obtained following the co-application of OVA and dexamethasone, which reduced goblet cells hyperplasia but did not affect smooth muscle thickness [41], [42]. These observations again demonstrate that slowly progressing remodelling features are more resistant to therapeutic interventions.

Our study also expands on works of Kumar and Herbert in which the authors showed that dexamethasone treatment resulted in reduced lung inflammation and collagen deposition [43], [44], by investigating airway inflammation and remodelling over a longer treatment period and by maintaining allergen challenge after the cessation of ICS. In a study by Southam *et al*. the simultaneous removal of both the allergen and ICS resulted in a marked rebound of goblet cell hyperplasia, which was most apparent after prolonged co-application of budesonide and allergen [30]. Interestingly a minimum of six weeks of concurrent budesonide/ICS administration was required to confer this rebound effect. In our study the continuation of allergen challenge after the discontinuation of ICS resulted in slightly increased eosinophil counts but did not affect goblet cell numbers or other remodelling characteristics. An important difference between these studies was that we maintained allergen challenge after cessation of ICS, a situation which reflects a non-compliant patient.

The disparity between ICS effects in acute and chronic asthma supports the concept that there is a shift in immune responses throughout disease progression in allergic asthma [3]. Therefore, the same therapy could confer different efficacy because of variability in the immune response pattern of different asthma patients. ICS are highly effective in reducing allergen induced eosinophilia and consequently in treating acute experimental asthma in which the eosinophils are the dominant cell type [45]. However, in chronic asthma phenotypes, which exhibit decreased eosinophils counts, other inflammatory cells have a more predominate role and are consequently less responsive to corticosteroid therapy.

In conclusion, using a chronic model of experimental asthma we have shown that continuous allergen exposure in mice induces reversible airway remodelling. Treatment of established inflammation and remodelling can be partially accomplished with corticosteroids, however, most prominent beneficial effects are observed by allergen avoidance. This model offers new opportunities to further delineate the cellular and molecular signalling pathways that contribute to the transition from the acute to the chronic phenotype, and to elaborate the pathways of normal repair and structural reorganisation.

## Supporting Information

Figure S1 **Prolonged allergen challenge results in decreased BALF inflammatory cell recruitment.** Differential cell counts from chronic allergic inflammation model following allergen challenge for up to 18 weeks, 6–8 animals per group, *p≤0.05, ***p≤0.001.
(TIFF)

Figure S2 **Inhaled corticosteroids attenuates features of acute airway inflammation.** (A) Acute airway inflammation was generated in mice via intraperitoneal (i.p.) injection of OVA conjugated to Alum and subsequently challenged with PBS or OVA for three days with or without the prior treatment with inhaled corticosteroids (ICS). Mice were analysed after 48 hrs for; (B) total BALF cells, (C) eosinophils, (D) lymphocytes, (E) neutrophils, (F) macrophages and (G) fraction of basal membrane covered by goblet cells. The results are presented as box and whiskers-plots and show mean and percentiles of 6–8 animals per group, *p≤0.05, **p≤0.01, ***p≤0.001. (BM  =  basement membrane).
(TIFF)
