# Supplementary figures and images for: Compartmental and Temporal Dynamics of Chronic Inflammation and Airway Remodelling in a Chronic Asthma Mouse Model
Source: PLoS One. 2014 Jan 21;9(1):e85839. doi: 10.1371/journal.pone.0085839 (PMC3897544; doi:10.1371/journal.pone.0085839)

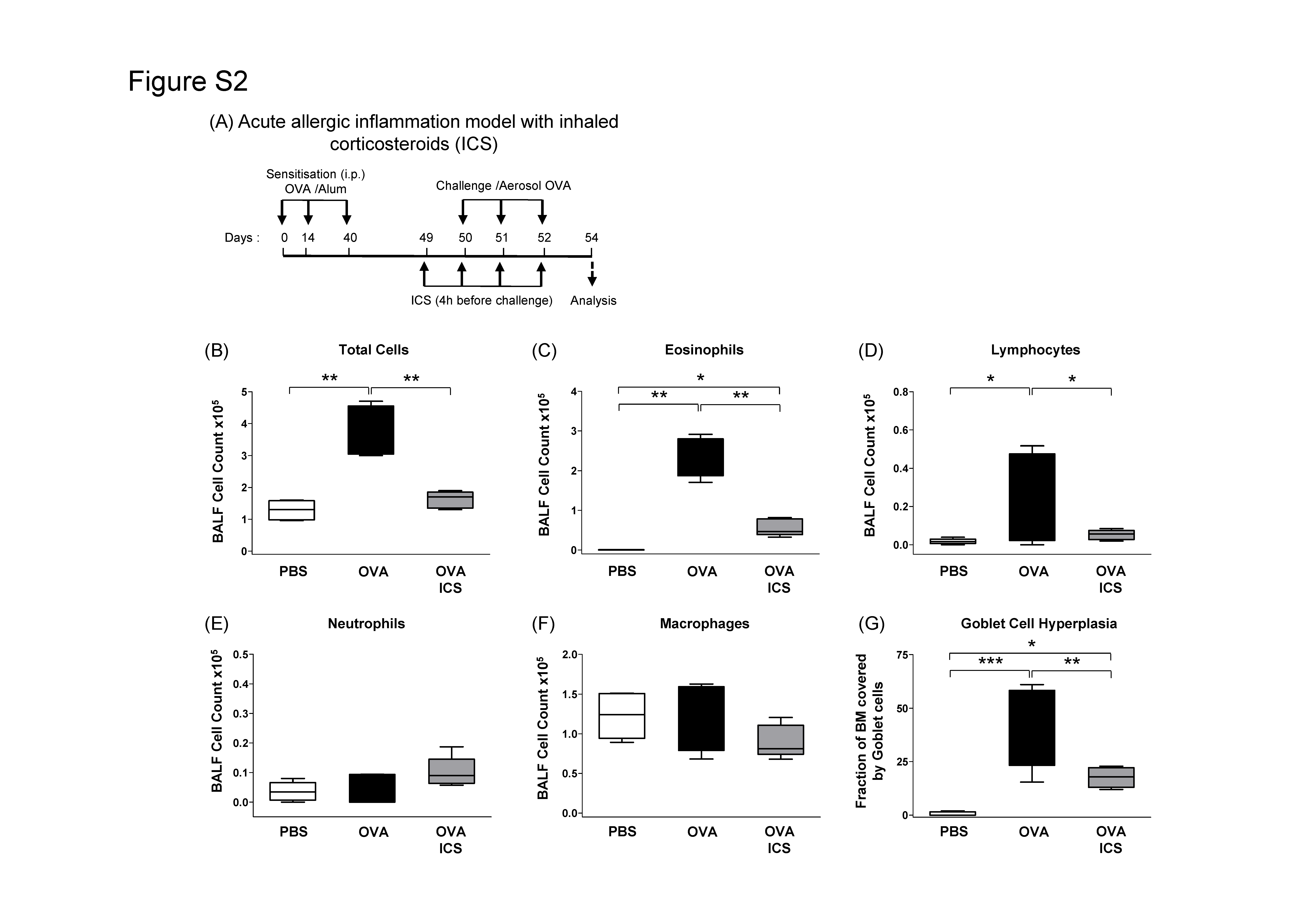

Supplement: Figure S2 — Inhaled corticosteroids attenuates features of acute airway inflammation. (A) Acute airway inflammation was generated in mice via intraperitoneal (i.p.) injection of OVA conjugated to Alum and subsequently challenged with PBS or OVA for three days with or without the prior treatment with inhaled corticosteroids (ICS). Mice were analysed after 48 hrs for; (B) total BALF cells, (C) eosinophils, (D) lymphocytes, (E) neutrophils, (F) macrophages and (G) fraction of basal membrane covered by goblet cells. The results are presented as box and whiskers-plots and show mean and percentiles of 6–8 animals per group, *p≤0.05, **p≤0.01, ***p≤0.001. (BM = basement membrane). (TIFF) [file pone.0085839.s002.tiff]
